# Prevalence of Subgingival *Aggregatibacter actinomycetemcomitans*: Descriptive Cross-Sectional Study

**DOI:** 10.3390/pathogens13070531

**Published:** 2024-06-24

**Authors:** Nabil Khzam, Omar Kujan, Dorte Haubek, Aysen Arslan, Anders Johansson, Jan Oscarsson, Zeinab Razooqi, Leticia Algarves Miranda

**Affiliations:** 1 UWA Dental School, The University of Western Australia, Nedlands, WA 6009, Australia; nokhzam@gmail.com (N.K.); leticia.algarvesmiranda@uwa.edu.au (L.A.M.); 2 NK Periodontics, Specialist Periodontal Private Practice, Applecross, WA 6152, Australia; 3 Jammerbugt Municipal Dental Service, Skolevej 1, DK-9460 Brovst, Denmark; dorte.haubek@outlook.dk; 4 Department of Odontology, Umeå University, 901 87 Umeå, Sweden; aysenarslan2003282@gmail.com (A.A.); jan.oscarsson@umu.se (J.O.); zeinab.razooqi@umu.se (Z.R.)

**Keywords:** *A. actinomycetemcomitans*, periodontitis, severity, new classification, extent, prevalence

## Abstract

This paper aims to investigate the presence of *Aggregatibacter actinomycetemcomitans* and to assess potential indicators of the risk of severe form(s) of periodontitis. A descriptive cross-sectional study of 156 consecutive patients with periodontitis was conducted. Subgingival plaque samples were collected from the participants. The identification of *A. actinomycetemcomitans* was performed using quantitative polymerase chain reaction. A descriptive analysis, a chi-square test, and a binary logistic regression statistical evaluation were performed. The prevalence of *A. actinomycetemcomitans* in this population of 156 participants was 17.30% (27 patients). The prevalence of stage-III periodontitis was 75.6% and greater in older men, while the prevalence of stage-IV periodontitis was 22.4% and greater in younger women. We observed a significant relation between the risk of severe periodontitis (stage-IV) and poor oral hygiene (*p* = 0.006), attendance at dental appointments (*p* ≤ 0.001), and familial history of periodontitis (*p* = 0.032). In conclusion, twenty-seven individuals were positive for *A. actinomycetemcomitans*. Poor oral hygiene, family history of periodontitis, and irregular attendance at dental appointments were identified as potential risk factors for severe periodontitis in this cohort.

## 1. Introduction

Periodontitis is a multifactorial oral disease; it affects the attachment apparatus that surrounds and supports the teeth. In addition, periodontitis is associated with chronic illnesses, such as diabetes mellitus, cardiovascular diseases, bone-related diseases, and others [1]. It is extremely important to understand the relationship between oral health and disease, as this clearly pinpoints the underlining genetics, microbial diversity, and functional activity. The oral microbiome is constantly under pressure from external insults and foreign substances, which could disrupt the balance between the microbial challenge and the host immune response, leading to disease [2]. There is evidence implicating *A. actinomycetemcomitans* as one of the key pathogens in periodontitis [3]. This sophisticated opportunistic pathogen of humans that is present in the oral cavity can destroy human leukocytes through the expression of exotoxins, leukotoxin LtxA, and the cytolethal distending toxin (Cdt) [4,5]. There are multiple lines of evidence that implicates *A. actinomycetemcomitans* in oral and systemic disease. Clinical, microbiological, genetics, and experimental data link the initiation, progression, and recurrence of periodontitis to *A. actinomycetemcomitans* [6,7]. Furthermore, *A. actinomycetemcomitans* has a critical role in the diagnosis and vaccination process in periodontitis [8,9]. The percentage of patients carrying *A. actinomycetemcomitans* and its strains seems to vary significantly between populations in various geographic areas worldwide and between ethnic groups [10]. It has been shown in subgingival samples from 1449 Swedish periodontitis patients that age is an important parameter for the prevalence of *A. actinomycetemcomitans* [11]. The prevalence and proportion of this bacterium was higher in young patients compared with that in older patients. Another study that compared periodontitis patients in Spain and the Netherlands found a difference in *A. actinomycetemcomitans* presence (23% versus 3%) [12]. The presence of *A. actinomycetemcomitans* has not been reported before in the Western Australian population.

In 2018, the ‘World of Periodontology’ came together from both sides of the Atlantic to propose a new classification of periodontal and peri-implant diseases and conditions [13]. However, there are hardly any epidemiological studies that adopted the data that this classification contains. Using the new classification in a future study is very important in establishing a unified diagnosis and treatment pathways of periodontitis.

The national survey of adult oral health of Australians was conducted between 2004 and 2006, in which 14,123 people aged 15–97 years old were interviewed and 5505 people were dentally examined. One in five Australian adults had moderate (20.5% of people) or severe (2.4% of people) periodontitis [14]. The case definition followed that in Beck‘s study [15]. The national study of adult oral health in Australia was published in 2019 [16]. In both Australian surveys, periodontitis was strongly associated with age, males, less schooling, lack of dental insurance, those eligible for public dental care, and people who visited the dentist for treatment of urgent dental problems.

This study aimed to investigate the carrier rate of the periodontal pathogen *A. actinomycetemcomitans* in subgingival dental plaque in a periodontitis cohort of Western Australians using quantitative real-time polymerase chain reaction (qPCR). The secondary aim was to assess potential indicators of the risk of severe form(s) of periodontitis in this group. 

## 2. Methods

### 2.1. Research Subjects

A descriptive cross-sectional study was conducted on 156 consecutive patients with periodontitis, aged 18 years and older, who were recruited from two private periodontal practices in Perth city, Western Australia, between June and November 2022 to take part in the present study. 

To be included in this study, the participants had to be from Western Australia, be 18 years and older, and not be taking any medication known to affect the periodontal tissue (antibiotics, host modulation agents, and pain killers). Participants were excluded from the study if they had any of the following within 3 months’ time of starting this study: teeth scaling, course of antibiotics, periodontal treatment, and acute periodontal infection. The patients answered a questionnaire about their biodata (age, gender, origin, level of education, residence, occupation, marital status, oral hygiene practice, last dental visit, para-functional habit(s), family history of periodontitis, smoking status, dental anxiety, and overall health). 

### 2.2. Clinical Examination

Periodontal and radiographic examinations were carried out by one experienced periodontist (N.K.). In each patient, the examination included measurement and scoring of a series of different parameters characterizing periodontitis. All surfaces of all teeth available (except wisdom teeth) were examined using an automated Florida probe system (Florida Probe Corporation, Gainesville, FL, USA). An orthopantomogram (OPG) was used to calculate the radiographic data. The Veraview X800 software program (J. MORITA MFG. CORP. Kyoto, Japan) was used to measure the bone loss in millimetres (bone loss amount in percentage of the root length) [13]. The collected periodontal clinical data included bleeding upon probing (BoP) [17], suppuration (present/absent), number of missing teeth, reason for tooth loss (periodontal/non-periodontal), plaque index (PI) [17], bone loss/age [13], bone loss [13], vertical bone loss (present/absent), periodontal pocket depth (PD) measured as the distance in millimetres from the free gingival margin to the bottom of the pocket, gingival recession measured as the distance from the cementoenamel junction to the free gingival margin, and clinical attachment loss (CAL). Three categories of PD and CAL were recorded [13]. 

### 2.3. Case Definition of Periodontitis

Cases with periodontitis were defined according to the 2018 classification of periodontal and peri-implant diseases and conditions [13]. 

### 2.4. Subgingival Plaque Sample Collection

In each patient, subgingival plaque samples were collected. The pooled subgingival plaque samples were collected using a sterile universal mini-curette. For each patient, subgingival plaque samples were collected from four diseased and four healthy sites (two samples per quadrant). The total number of samples collected was 1248 subgingival plaque samples; these samples were pooled into 156 samples (one sample per patient). The subgingival plaque samples were collected in sterile ice-chilled 5.0 mL Eppendorf tubes (Eppendorf South Pacific Pty, Ltd., Macquarie Park, NSW, Australia). A courier with specialized freezing equipment was used for the transportation of the coded subgingival plaque samples; all samples were sent from NK Periodontics practices in Western Australia to the Dental School, Department of Odontology, Umeå University, Sweden, for subsequent processing and analysis. 

### 2.5. Bacterial DNA Extraction

For DNA isolation from the plaque samples, a GXT NA Extraction Kit^®^ (Hain Lifescience, GmBH, Nehren, Germany) and an Arrow automated extraction instrument (Liaison IXT, DiaSorin Ltd., Fort Henry, Ireland) were used, following the procedures described earlier [18].

### 2.6. DNA Quantification

The amount of total extracted DNA in each plaque sample was quantified using a NanoDrop (Thermo Fisher, Waltham, MA, USA) instrument. For quantification of *A. actinomycetemcomitans* loads, suspensions of the reference strain HK1651 were treated as described and used for standard curves, i.e., by being serially diluted [18].

A Corbett Research Rotor-Gene 6000 Rotary Analyze instrument (QIAGEN, Valencia, CA, USA) was used for the quantification of the total concentration of *A. actinomycetemcomitans* loads in the samples, using qPCR. The cycling conditions used were according to the Kirakodu method [19]. The oligonucleotide primers used were as follows: forward 5′-CTAGGTATTGCGAAACAATTTG-3′ and reverse 5′-CCTGAAATTAAGCTGGTAATC-3′. A load of 100 *A. actinomycetemcomitans* cells per ml of sample was set as a positive result regarding the presence of this bacterium. The DNA concentration of *A. actinomycetemcomitans* in each sample was determined from duplicates. All laboratory work was carried out by an experience team at Umeå University (A.A., J.O., and Z.R.).

### 2.7. Measurement Reliability of the Examiner and Reproducibility

To determine the intra-examiner consistency, the principal investigator (N.K.) re-examined ten patients twice, fourteen days a part. A Kappa value of 0.87 was obtained for PD measurements in this study, with a score of ≤0.4 being considered poor to fair agreement, 0.41–0.60 being moderate agreement, 0.61–0.80 being substantial agreement, and 0.8–1.00 being excellent agreement. 

### 2.8. Statistical Analysis

The collected data were analysed using IBM SPSS Statistics software program version 29.0 (SPSS Inc., Chicago, IL, USA). The primary outcomes were the presence of *A. actinomycetemcomitans* and the severity of periodontitis. A descriptive analysis was performed, evaluating the quantitative and qualitative variables, and presented in tables. Furthermore, a chi-square test was carried out to evaluate the relationships between gender, level of education, marital status, oral hygiene methods, dental visits, smoking, and occupation. More analyses were conducted to evaluate the relationships between different levels of periodontitis; *A. actinomycetemcomitans* presence; and other potential risk factors like age, gender, overall health, oral hygiene methods, frequency of dental visits, marital status, and smoking status. The confidence level was set at 95%. To assess the risk of an advanced form of periodontitis with the presence of the different potential risk factors evaluated, the methodology of binary logistic regression was used. The significance level used was 5%.

The sample size of the present study was calculated by averaging the prevalence of *A. actinomycetemcomitans* from studies of similar cohorts. We estimated the average prevalence of the present study to be around 10% in order to achieve a 95% confidence interval with 5% precision; thus, we required a sample size of at least 139 participants.

### 2.9. Ethical Considerations

Those who agreed to participate in this study signed an informed consent form. All patients’ data were entered into a cloud-based software program using coded numbers to protect their privacy. Approval to conduct the current study was given by the Human Ethics, Office of Research at The University of Western Australia (2022/ET000252). 

## 3. Results 

### 3.1. Levels of A. Actinomycetemcomitans

*A. actinomycetemcomitans* was present in 27 (17.30%) of the participants, and their levels of this bacterium in the analysed samples are presented in Table 1, which also demonstrates additional characteristics of the *A. actinomycetemcomitans*-positive patients. Table 2 shows an association between the detection of *A. actinomycetemcomitans* and the other variables. The mean DNA concentration of *A. actinomycetemcomitans* was 106.11 ng/μL (SD ± 20.06) in *A. actinomycetemcomitans*-positive patients (n = 27) compared to 8.50 ng/μL (SD ± 16.55) in the *A. actinomycetemcomitans*-negative patients (n = 129). 

### 3.2. Sociodemographic Data

One hundred and fifty-six patients were included in the present study. Of these, 83 (53.2%) were females and 73 (46.8%) were males. The age ranged between 26 and 86, with a mean of 54.03 years ± 12.13 S.D (standard deviation). Of the patients, 83 were of non-Australian origin or were born overseas (53.2%). A total of 106 of the patients were married (67.9%). A total of 100 (64.1%) patients were from Perth city, with the remaining 56 (35.9%) residing outside Perth city. In total, 120 of the patients were older than 40 years old (76.9%) (Table 3). The chi square test showed no statistically significant association between gender and level of education, marital status, and occupation. The chi square test also showed no statistical significance between marital status and level of education (Table 4). 

### 3.3. Tooth Loss Data

Two males (2.7%) had no tooth loss, and another thirty-one males (42.5%) had lost four teeth or less compared to forty males (54.8%) who lost five or more teeth. Periodontitis was the reason for tooth loss in 42.5% of the males. Three females (3.6%) had no tooth loss, and thirty-eight females (45.8%) who had lost four teeth or less compared to forty-two (50.6%) who had lost five teeth or more. Periodontitis was the reason for tooth loss in 44.6% of the females.

A total of fourteen (38.9%) of this age group lost their teeth due to periodontitis compared to twenty-two (61.1%) who lost their teeth due to other causes. Two patients (5.6%) of this group had no tooth loss compared to twenty-eight (77.8%) and six patients (16.7%) who lost four or less teeth and five or more teeth, respectively. In the older age group, fifty-four patients (45%) lost their teeth due to periodontitis. Of this age group, three patients (2.5%) had no tooth loss, forty-one (34.2%) lost four or less teeth, and seventy-six (63.3%) lost five or more teeth. 

### 3.4. Potential Risk Factors for Periodontitis

On assessment of the known risks factors for periodontitis, 15 of the patients (9.6%) gave a medical history of managed systemic illnesses and treatment (such as diabetes mellitus type II, osteoporosis, and cancer therapy). Twenty of the patients (12.8%) were smokers. 

A total of 118 of the patients (75.6%) were regular attenders at their general dentist for regular check-up and dental hygienist appointments compared to 38 patients who would only visit the dentist if they have issues like dental pain (24.4%). In total, 65.4% of the patients (n = 102) had good oral hygiene, with daily toothbrushing and flossing, compared to 34.6% of the patients (n = 54), who only brush their teeth. In Table 5, oral hygiene method (toothbrushing alone versus toothbrushing and flossing) is reported in relation to sociodemographic variables. Using a chi square test, there was a statistically significant difference between oral hygiene methods and gender, with more female patients both toothbrushing and flossing compared to male patients (X^2^ = 5.154, *p* = 0.02). There was a statistically significant difference between oral hygiene method and visits to the dentist, meaning that patients using combined toothbrushing and flossing were the ones who had high attendance at their general dentist appointments (X^2^ = 66.78, *p* ≤ 0.001). Non-smokers used the combined toothbrushing and flossing methods to look after their gum health significantly more often than smokers (X^2^ = 9.35, *p* = 0.004). There was no statistically significant difference between oral hygiene methods and the level of education and marital status. 

A total of 84 of the patients had a family history of periodontitis (53.8%), compared to 60 patients (38.5%) who reported no family history of periodontitis, while 12 patients (7.7%) did not know of any family history of periodontitis (2 patients were adopted).

### 3.5. Clinical Outcomes 

Table 6 reveals the distribution of the mean, standard deviation, and percentages of PD, CAL, BoP, PI, suppuration presence, bone loss/age, presence of bone loss, and presence of vertical bone loss according to gender, age, and *A. actinomycetemcomitans* presence. The results reveal that females have a higher value than males in terms of mean PD and CAL and that high mean values are more common in younger patients. Both PI and BoP scores were higher for men than women, while bone loss scores were higher for women than men.

### 3.6. Relationship between Periodontitis and Potential Risk Factors

Regarding the disease staging, 118 of the patients (75.6%) had stage-III periodontitis, whereas 35 (22.4%) had stage-IV periodontitis. Regarding the disease grade, 114 (73.1%) of the patients had grade-B periodontitis compared to 42 (26.9) of the patients who had grade-C periodontitis. In terms of disease extent, 130 patients (83.3%) had a generalized disease. As Table 7 shows, an association was found between the severity of periodontitis and oral hygiene protocol. The patients who only brushed their teeth had more risk of developing an advanced form of periodontitis compared to those who used both toothbrushing and flossing (X^2^ = 10.15, *p* = 0.006). Also, another relationship between periodontitis and irregular attendance at dental appointments was found, with the more advanced form of periodontitis associated with those who only attend dental practices for emergency dental treatment (X^2^ = 14.78, *p* ≤ 0.001). 

The results of the binary logistic regression (with a probability for entry = 0.05 and for removal = 0.10), including variables with a potential relation to the presence of severe forms of periodontitis, are presented in Table 8. The Omnibus Tests of Model Coefficients are significant. This shows that there is a significant improvement in fit, as compared to the null model; hence, the model shows a good fit (*p* = 0.024). In other words, the model describes the included data very well. The Hosmer and Lemeshow Test is also a test for model fit. This statistical test indicates a poor fit if the significance value is less than 0.05. Here, the model adequately fits the data. Hence, there is no difference between the observed and predicted models (*p* = 0.579). 

An odds ratio was obtained for the following variables: family history of periodontitis B = −0.79, OR = 0.45, meaning that the patients with a history of periodontitis had double the risk of having a severe form of periodontitis compared to those with no family history of periodontitis. For the variable dental visit attendance, B = 1.40, OR = 4.07, meaning that the patients who were irregular attenders at their dental appointments were four times more likely to have an advanced form of periodontitis. 

## 4. Discussion

*A. actinomycetemcomitans* is associated with periodontitis and systemic diseases, such as infective endocarditis, bacteremia, meningitis, and skin infection [20]. In the present work, the *A. actinomycetemcomitans* prevalence was 17.30%. The prevalence of *A. actinomycetemcomitans* is high in Asian and African populations but is significantly lower in Europe [21,22,23,24,25]. Our results are comparable to previous findings in an European population [26,27,28]. In one study in Finland, *A. actinomycetemcomitans* was positive in 13% of the population [25]. The prevalence in Switzerland and Italy was found to be 20% and 16%, respectively [29,30]. This relatively low representation of *A. actinomycetemcomitans* may be a representation of the European settlements in Western Australia.

Our data showed more risk of severe periodontitis with age, especially with young females. This is in contrast with other studies that showed an increase in the severity of periodontitis in elderly males [31,32,33,34]. However, those studies used a half mouth recording periodontal protocol, which can contribute to disease underestimation and inaccuracy. Our data revealed that the prevalence of stage-III periodontitis was 75.6% and great in older men, while the prevalence of stage-IV periodontitis was 22.4% and greater in younger women. A study by Relvas and co-authors found that the prevalence of stage-III periodontitis was 51.2% and greater in older males, while stage-IV periodontitis was 30.4% and again greater in older males [34]. Regarding the extent of periodontitis in our study, there was a higher prevalence of the generalized form (83.3%), which is in agreement with other studies [31,32,33,34]. It is worth mentioning that our study is the only one that has revealed an incisor–molar pattern of periodontitis extension using the 2018 classification [13]. 

Regarding clinical variables, the mean PD and CAL were higher in females compared to males and higher in the younger age group compared to the older age group. These figures were in line with other studies, but these averages were higher in our study. One reason is that the full mouth examination periodontal protocol that we used likely limited underestimation of the disease and contributed to more accuracy [33,34,35]. 

The subjects with an oral hygiene routine consisting of toothbrushing only and no flossing (X^2^ = 10.15, *p* = 0.006), and those who only attended dental appointments for emergencies (X^2^ = 14.78, *p* ≤ 0.001, B = −1.406, OR = 4.07) were found to be at a high risk of having severe periodontitis. The same apply to the patients with a familial history of periodontitis (B = −0.79, OR = 0.45).

Our study has a number of strengths and weakness, like any other study. We are the first study to explore the presence of *A. actinomycetemcomitans* in a cohort of Western Australia. We are also the first study to calculate the grading of periodontitis according to the 2018 classification. In our study, 114 (73.1%) of the patients had grade-B periodontitis compared to 42 (26.9%) of the patients having grade-C periodontitis. Missing teeth due to periodontal disease is a very vital piece of information and can be used to identify the stages of periodontitis. In our study, more teeth were lost to periodontitis in stage-III periodontitis cases than any other stages. One explanation for this could be due to the large number of patients in this group. 

The downsides of our study are the lack of control groups (healthy periodontia), the inability to detect a cause–effect relationship, and the inability to guarantee results representation due to the snapshot nature. All patients were referred to a periodontal practice, so most of them were periodontally unstable. The convenience sampling method utilized indicates that periodontitis experience in the participants was more likely to be higher than in a randomly selected study population.

## 5. Conclusions 

This study revealed a relatively low presence of *A. actinomycetemcomitans* in a cohort of Western Australia. The patients with a family history of periodontitis had double the risk of having a severe form of periodontitis compared to those with no family history of periodontitis. In addition, the patients who were irregular attenders at their dental appointments were four times more likely to have an advanced form of periodontitis compared to regular attenders. Poor oral hygiene was also identified as a potential risk factor for periodontitis in this cohort.

## Figures and Tables

**Table 1:** *A. actinomycetemcomitans*-positive patients and their characteristics.

Pt/Variable	Sex	Age/Years	Origin	Stage/Grade	Status of Health	*Aa* Levels ng/μL	Status
Pt 1	F	70	Philippines	III/B	-	848,041	M
Pt 2	M	48	Australia	IV/C	-	22,379	M
Pt 3	F	48	Australia	III/B	-	10,401	M
Pt 4	M	40	New Zealand (Māori)	IV/C	S	1719	M
Pt 5	M	49	New Zealand (Māori)	III/B	-	1680	M
Pt 6	F	56	Thailand	III/B	DM-II	1448	M
Pt 7	M	54	New Zealand (Māori)	III/B	-	303	M
Pt 8	M	68	Australia	IV/C	-	365,681	M
Pt 9	M	34	Tonga	IV/C	DM-II	851	M
Pt 10	F	56	UK	III/B	-	153	M
Pt 11	F	65	New Zealand (Māori)	IV/C	-	10,651	S
Pt 12	F	40	Australia	III/B	-	577	M
Pt 13	M	39	United Kingdom	III/B	-	153	S
Pt 14	F	62	Kenya	III/B	-	381,842	M
Pt 15	F	36	Australia	III/B	-	4624	M
Pt 16	M	40	Australia	III/B	-	340	S
Pt 17	M	57	Samoa	III/B	S	56,334	M
Pt 18	F	47	Palestine	III/B	-	46,227	M
Pt 19	M	40	Australia	III/B	-	184,822	S
Pt 20	M	75	Italy	III/B	-	132	M
Pt 21	M	56	South African	III/B	-	479,910	M
Pt 22	M	52	Sri Lanka	III/B	-	5163	M
Pt 23	F	60	South Africa	IV/C	-	254,437	S
Pt 24	F	40	Ireland	III/B	-	81,934	M
Pt 25	M	68	Australia	III/B	DM-II	3598	S
Pt 26	M	33	Australia	III/B	S	101,611	M
Pt 27	M	66	Australia	III/B	DM-II	138.5	M

Pt: patient; sex: M: male/F: female; S: smoking; DM-II: type II diabetes mellitus; status: S: single/M: married.

**Table 2:** Presence of *A. actinomycetemcomitans* based on sociodemographic and other variables.

Variable		*Aa*		Test	DF	*p*-Value
		Present N (%)	Absent N (%)			
Age	≤40 years old >40 years old	9 (33.3) 18 (66.7)	27 (20.9) 102 (79.1)	1.935	1	0.164
Gender	Male Female	16 (59.3) 11 (40.7)	57 (44.2) 72 (55.8)	2.037	1	0.153
Origin	Australian Non-Australian	10 (37.0) 17 (63.0)	63 (48.8) 66 (51.2)	1.249	1	0.264
Smoking	Smoker Non-smoker	3 (11.1) 24 (88.9)	17 (13.2) 112 (86.8)	0.085	1	0.770

N: number; %: percentage; DF: degree of freedom.

**Table 3:** Study population characteristics.

Characteristics	Mean ± SD/N
Patient’s number	156
Age (years)	54.03 ± 12.13
Younger/Older (more than 40 years old)	(36/120)
Gender (male/female)	73/83
*A. actinomycetemcomitans* (positive/negative)	27/129
Origin (Australian/overseas)	73/83
Marital status (single/married)	50/106
Residence (Perth/rural)	100/56

N: number; SD: standard deviation.

**Table 4:** The relationship between sociodemographic characteristics and gender/marital status.

Variable		Gender			
				X^2^	*p*-Value
		Male N (%)	Female N (%)		
Education	Low	64 (87.7)	74 (89.2)	0.08	0.77
	High	9 (12.3)	9 (10.8)		
Marital status	Married	53 (72.6)	53 (63.9)	1.36	0.24
	Single	20 (27.4)	30 (36.1)		
Occupation	Working	51 (69.9)	53 (63.9)	0.63	0.42
	Retired	22 (30.1)	30 (36.1)		
Marital status					
		Married N (%)	Single N (%)		
Education	Low	91 (85.8)	47 (94)	2.21	0.13
	High	15 (14.2)	3 (6)		

N: number; %: percentage.

**Table 5:** Oral hygiene methods in relation to sociodemographic and other variables.

Variables		Oral Hygiene Method		X^2^	*p*-Value
		Toothbrushing only N (%)	Toothbrushing and Flossing N (%)		
Gender	Male	32 (59.3)	41 (40.2)	5.15	0.02
	Female	22 (40.7)	61 (59.8)		
Education	Low	50 (92.6)	88 (86.3)	1.38	0.24
	High	4 (7.4)	14 (13.7)		
Marital status	Married	33 (61.1)	73 (71.6)	1.77	0.18
	Single	21 (38.9)	29 (28.4)		
Dental visits	Yes	20 (37)	98 (96.1)	66.79	<0.001
	No	34 (63)	4 (3.9)		
Smoking	Yes	13 (24.1)	6.9 (20)	9.35	0.004
	No	41 (75.9)	95 (93.1)		

N: number; %: percentage.

**Table 6:** Summary of the distribution of categorial variables for periodontal parameters related to gender, age, and presence of *A. actinomycetemcomitans* presence.

Variables		Mean PD	PD ≤ 4 mm (%)	PD = 5 (%)	PD ≤ 6 (%)	Mean CAL	CAL 1–2 (%)	CAL 3–4 (%)	CAL ≥ 5 (%)	BoP (%)	PI (%)	Pus (%)	BL/Age > 1 (%)	BL > 33 (%)	V-BL (%)
Sex	M	5.13 (1.97)	42.6	13.2	44.2	5.55 (1.85)	3.9	25.4	70.7	52.6	57.1	3.5	44.6	46.1	24.2
	F	5.30 (2.12)	39.5	12.9	47.6	5.62 (2.05)	4.8	27.9	67.3	46	48.3	5.5	58.3	61	45
Age	≤40 Yrs	5.36 (2.11)	37.6	13.3	49.1	5.71 (1.99)	4.6	25.2	70.3	50.6	54.3	4.5	50.7	52.9	38
	>40 Yrs	5.14 (2.39)	42.8	13	44	5.51 (1.95)	4.7	27.7	67.6	48	51.2	4.4	47.9	54.9	34.4
*Aa*	Present	5.29 (1.98)	37.4	11.9	50.7	5.60 (1.95)	5.2	25.0	69.8	48.4	51.6	6.9	51.6	48.6	36.9
	Absence	5.20 (2.07)	41.8	13.4	44.8	5.57 (1.97)	4.3	27.4	68.3	49.2	52.5	4.2	52	54.5	35

%: percentage; ±SD: standard deviation; M: male; F: female; PD: pocket depth; CAL: clinical attachment loss; BoP: bleeding upon probing; PI: plaque index; Yrs: years; BL: bone loss; V-BL: vertical bone loss.

**Table 7:** Periodontitis and potential risk factors.

Variables		Periodontitis					
		Stage-II N (%)	Stage-III N (%)	Stage-IV N (%)	Test	DF	*p*-Value
Age	≤40 years old	1 (2.8)	22 (61.1)	13 (36.1)	X^2^ = 5.38	2	0.068
	>40 years old	2 (1.7)	96 (80)	22 (18.3)			
Gender	Male	2 (2.7)	57 (78.1)	14 (19.2)	X^2^ = 1.23	2	0.540
	Female	1 (1.2)	61 (73.5)	21 (25.3)			
Smoking	Non-smoker	2 (1.5)	106 (77.9)	28 (20.6)	X^2^ = 3.48	2	0.175
	Smoker	1 (5)	12 (60)	7 (35)			
Oral Hygiene	Toothbrushing only	1 (1.9)	33 (61.1)	20 (37)	X^2^ = 10.15	2	0.006
	Toothbrushing + flossing	2 (2)	85 (83.3)	15 (14.7)			
Systemic diseases	Yes	1 (6.7)	11 (73.3)	3 (20)	X^2^ = 1.99	2	0.368
	No	2 (1.4)	107 (75.9)	32 (22.7)			
Dental visits	Irregular	1 (2.6)	20 (52.6)	17 (44.7)	X^2^ = 14.78	2	<0.001
	Regular	2 (1.7)	98 (83.1)	18 (15.3)			
Marital status	Single	2 (4)	36 (72)	12 (24)	X^2^ = 1.86	2	0.39
	Married	1 (0.9)	82 (77.4)	23 (21.7)			

N: number; %: percentage.

**Table 8:** Results of the binary logistic regression analysis.

Variable						95% CI for OR	
	B	S.E.	Wald	*p*	OR	LL	UL
Family history of periodontitis	−0.799	0.372	4.624	0.032	0.450	0.217	0.932
Dental visit attendance	1.406	0.715	3.870	0.049	4.079	1.005	16.552

B: estimates for the slope coefficients of the univariate logistic regression model containing only this variable; S.E: estimated standard error for the estimated coefficient; *p*: value associated with the statistical coefficient test; OR: estimated odds ratio; CI: confidence interval of 95% for odds ratio; LL: lower limit; UL: upper limit.

## Data Availability

The data supporting this study’s findings are available by emailing the corresponding author.
